# Advances in Molecular Diagnostics

**DOI:** 10.1155/2013/172521

**Published:** 2013-05-27

**Authors:** Tavan Janvilisri, Arun K. Bhunia, Joy Scaria

**Affiliations:** ^1^Department of Biochemistry, Faculty of Science, Mahidol University, Bangkok 10400, Thailand; ^2^Department of Food Science and Department of Comparative Pathobiology, Purdue University, West Lafayette, IN 47907, USA; ^3^Department of Population Medicine and Diagnostic Sciences, College of Veterinary Medicine, Cornell University, Ithaca, NY 14850, USA

People at present day are facing serious global challenges in healthcare from emerging and reemerging diseases. Research in molecular diagnostics has provided us with the better understanding of molecular processes affecting human health, and diseases and the tools derived thereof are becoming the standard of care for treatment of several diseases. The availability of new sequencing methods, microarrays, microfluidics, biosensors, and biomarker assays has made a shift toward developing diagnostic platforms, which stimulates growth in the field by providing answers to questions regarding diagnosis, prognosis, and best course of treatment, leading to improved outcomes and greater cost savings. It is equally important to identify and resolve existing challenges that impede the effective translation of validated diagnostic biomarkers from laboratory to clinical practice in order to see results from these efforts. 

This special issue contains nine articles, where one review article focuses on microvesicles as a potential biomarker for ovarian cancer, three papers are related to nucleic-acid-based detection of pathogens. Two papers focus on the development of detecting methods while another paper aims to evaluate the types of samples for diagnosis. Finally, two papers address the relationship between biomarkers and cancer. Thus, the papers in this special issue, representing a broad spectrum of experimental approaches and areas of investigation, demonstrate a wide array of molecular diagnostic research. This unique and informative collection of papers in “Advances in Molecular Diagnostics” showcases the identification and characterization of molecular biomarkers and the development of practical applications or methodologies for diagnosis and prognosis including the evaluation of the efficacy of various diagnostic platforms in both laboratory and clinical settings. There are also papers dealing with criteria of optimal detection methods in clinical samples, leading to a guideline as a tool for clinical guidance.

In “*Microvesicles as potential ovarian cancer biomarkers*,” I. Giusti et al. provided us a perspective review that summarizes the potential use of microvesicles released from tumor cells, especially regarding their microRNA profiles, as a novel molecular biomarker for ovarian cancer.

In “*Development of a broad-range 23S rDNA real-time PCR assay for the detection and quantification of pathogenic bacteria in human whole blood and plasma specimens*,” P. Gaibani et al. developed a new broad-range real-time PCR assay targeting the 23S *rDNA* gene that could detect the targeted bacterial 23S *rDNA* gene as low as 10 plasmid copies per reaction. This assay could allow us to quantify total bacterial DNA in the whole blood and plasma samples without the need for a precise bacterial species identification. 

In “*A pentaplex PCR assay for the detection and differentiation of Shigella species*,” S. C. Ojha et al. developed a pentaplex PCR assay for the simultaneous detection and differentiation of the *Shigella* genus and the three *Shigella* species responsible for the majority of shigellosis cases. The average detection of this PCR assay was 5.4 × 10^4^ CFU/mL, which is within the common detection limit for *Shigella*.

In “*Evaluation of multiplex PCR with enhanced spore germination for detection of Clostridium difficile from stool samples of the hospitalized patients,*” S. Chankhamhaengdecha et al. designed a new multiplex-PCR based assay for the detection of *C. difficile* and evaluated the sample processing steps prior to the multiplex PCR diagnosis from clinical stool samples. This enrichment multiplex PCR could be an alternative approach to enzyme immuno assays for rapid and cost-effective detection of *C. difficile*.

In “*Microsphere suspension array assays for detection and differentiation of Hendra and Nipah viruses,*” A. J. Foord et al. presented microsphere suspension array assays to simultaneously identify multiple separate nucleotide targets in a single reaction. The main goal of this research was to incorporate the Hendra and Nipah viruses microsphere as modules in multiplexed microsphere arrays. Their results were comparable to qPCR, indicating high analytical and diagnostic specificity and sensitivity.

In “*Development of a generic microfluidic device for simultaneous detection of antibodies and nucleic acids in oral fluids*,” Z. Chen et al. demonstrated a portable processing system for disposable microfluidic chips suitable for point-of-care settings in the diagnosis, detection, and confirmation of infectious disease pathogens. The HIV infection was used as a model to investigate the simultaneous detection of both human antibodies against the virus and viral RNA. 

In “*Urine cell-free DNA integrity as a marker for early prostate cancer diagnosis: a pilot study,*” V. Casadio et al. evaluated the potential use of urine cell-free DNA as a promising noninvasive marker for the early diagnosis of prostate cancer. The overall diagnostic accuracy was approximately 80%. The preliminary data in this paper could pave the way for confirmatory studies on larger case series.

In “*Development of a novel system for mass spectrometric analysis of cancer-associated fucosylation in plasma *α*1-acid glycoprotein,*” T. Asao et al. evaluated the fucosylated glycans as novel tumor markers that could be of clinical relevance in the diagnosis and assessment of cancer progression as well as patient prognosis. They also developed a novel software system for use in combination with a mass spectrometer to determine N-linked glycans in *α*1-acid glycoprotein that could be valuable for screening plasma samples to identify biomarkers of cancer progression based on fucosylated glycans.

In “*The associated ion between the VDR gene polymorphisms and susceptibility to hepatocellular carcinoma and the clinicopathological features in subjects infected with HBV,*” X. Yao et al. evaluated the possible association between the vitamin D receptor (VDR), single-nucleotide polymorphisms (SNPs), and hepatocellular carcinoma (HCC) in patients with chronic hepatitis B virus (HBV) infection and found that the C > T polymorphisms at FokI position in the *VDR* gene served as a potential biomarker for the risk and the disease severity of HCC in those infected with HBV.

Finally, we would like to thank the authors for their contributions in this special issue and all reviewers for critical review of the manuscripts.

*Tavan Janvilisri* *Arun K. Bhunia* *Joy Scaria*

